# Implementation and application of a multiplex assay to detect malaria-specific antibodies: a promising tool for assessing malaria transmission in Southeast Asian pre-elimination areas

**DOI:** 10.1186/s12936-015-0868-z

**Published:** 2015-09-04

**Authors:** Karen Kerkhof, Lydie Canier, Saorin Kim, Somony Heng, Tho Sochantha, Siv Sovannaroth, Inès Vigan-Womas, Marc Coosemans, Vincent Sluydts, Didier Ménard, Lies Durnez

**Affiliations:** Department of Biomedical Sciences, Institute of Tropical Medicine, Antwerp, Belgium; Department of Biomedical Sciences, University of Antwerp, Antwerp, Belgium; Molecular Epidemiology Unit, Institut Pasteur du Cambodge, Phnom Penh, Cambodia; National Centre for Parasitology, Entomology and Malaria Control, Phnom Penh, Cambodia; Infectious Diseases Immunology, Institut Pasteur de Madagascar, Antananarivo, Madagascar; Department of Biology, University of Antwerp, Antwerp, Belgium

**Keywords:** Malaria, Serological markers, Multiplex immunoassay, Cambodia

## Abstract

**Background:**

Epidemiological surveillance is a key activity in malaria control and elimination in low-transmission and pre-elimination settings. Hence, sensitive tools for estimating malaria force of infection are crucial. Serological markers might provide additional information in estimating force of infection in low-endemic areas along with classical parasite detection methods. Serological markers can be used to estimate recent, past or present malaria exposure, depending on the used markers and their half-life.

**Methods:**

An assay based on 14 *Plasmodium*-specific peptides, one peptide specific for *Anopheles gambiae* saliva protein and five *Plasmodium*-specific recombinant proteins was developed for the MAGPIX system, assessed for its performance, and applied on blood spots from 2000 individuals collected in the Ratanakiri Province, Cambodia.

**Results:**

A significant correlation for the use of 1000 and 2000 beads/antigen/well as well as for the monoplex versus multiplex assay was observed for all antigens (p < 0.05). For the majority of antigens, antigen-coupled beads were stable for at least 2 months. The assay was very reproducible with limited intercoupling, interplate and intraplate variability (mean RSD <15 %). Estimating seroconversion and seroreversion per antigen using reversible catalytic models and models allowing two seroconversion rates showed higher seroconversion rates in adults.

**Conclusion:**

The multiplex bead-based immunoassay was successfully implemented and analysis of field blood samples shows that changes detected in force of malaria infection vary according to the serological markers used. Multivariate analysis of the antibody responses and insights into the half-life of antibodies are crucial for improving the interpretation of these results and for identifying the most useful serological markers of past and recent malaria infection.

**Electronic supplementary material:**

The online version of this article (doi:10.1186/s12936-015-0868-z) contains supplementary material, which is available to authorized users.

## Background

Globally an estimated 3.4 billion people in 107 malaria-endemic countries are at risk of malaria, of which 1.2 billion at high risk [1] live mostly in the African region (47 %) and the Southeast Asian region (37 %). To control and eliminate malaria, WHO recommends a multi-pronged strategy, which includes vector control interventions, preventive therapy, diagnostic testing, treatment with quality-assured artemisinin-based combination therapy (ACT) as well as strong epidemiological surveillance [1]. Through upscaling of several elements of this strategy, many countries are on the verge of reaching pre-elimination. The low transmission rates in these areas pose considerable challenges for epidemiological surveillance [2], hindering the evaluation of new (vector) control tools necessary to reach elimination [1].

Detection of malaria-infected persons by microscopy, rapid diagnostic tests (RDTs) and even PCR lacks sensitivity because of low numbers of positive samples [3], representing a formidable logistical challenge due to the very big sample sizes required in surveillance and evaluation surveys. While parasite-prevalence (measured by microscopy, RDTs or PCR) provides a snapshot of the exposure to malaria, the use of serological markers can provide a picture of the malaria transmission over a prolonged period [3, 4]. Serology is based on the detection of antibodies (Abs) against antigens (Ags) of malaria parasites, which offers an advantage as anti-*Plasmodium* Abs can persist for months after infection. Therefore, these Abs have been suggested as indicators of malaria transmission [5–8], and are believed to be a better approach to determine past, recent and present malaria exposure [9]. Previous studies performed in low transmission settings, such as Cambodia, also suggest that serological assays are promising for indicating malaria transmission [3, 10–12].

Since the 1960s, serological markers detected by indirect immunofluorescence antibody tests (IFAT) were used to assess malaria transmission intensity and reductions in transmission [13]. This has proven to be a reliable and useful serological test for malaria in epidemiological surveys [5, 6, 14]. However, variation in source of Ags and the subjectivity of IFAT has led to this method falling out of favour [4]. Standardized tests based on recombinant Ags used in an enzyme-linked immunosorbent assay (ELISA) were therefore developed [4, 7–9]. However, an ELISA can only assess one marker at a time, making it labour intensive and time consuming when interested in multiple Ab responses. In the context of malaria elimination it will become essential to take into account individual variations in Ab responses, the occurrence of multiple malaria parasites [15], as well as to increase the probability of measuring changes in Ab responses by combining different markers. Recently, several multiplex assays that were testing for different serological markers in the same blood sample, were developed by different research teams based on the Luminex technology [15–18].

In this context, the general objective of this study was to implement an existing assay based on the Luminex technology for detection of Abs against malaria parasites in blood samples from Ratanakiri Province, Cambodia. This is the first and most extensive multiplex assay in malaria serology executed in the Southeast Asian region, including 20 Ags (recombinant proteins and peptides) directed against different specific malaria parasites. Furthermore, this study includes a detailed analysis on the stability of coated beads over time and the reproducibility of the beads coupling and immunoassay.

## Methods

### Samples

A positive control for the assay was prepared by pooling sera from four *Plasmodium falciparum*- and two *Plasmodium vivax*-infected patients from Ratanakiri Province in Cambodia. Dilutions of this positive control pool were prepared at 1:100, 1:400 and 1:1600 in PBS-CR (phosphate buffered saline, Charles River Laboratories Inc, MA, USA). These dilutions were used to assess the performance of the assay and as positive control samples in the immunoassay applied on field blood samples. The latter were collected in Ratanakiri Province, Cambodia, on filter papers through finger prick, during April–May 2012 (baseline survey of the MalaResT project, NCT01663831, that aims to evaluate the use of topical repellents, in addition to long-lasting insecticidal nets, on malaria prevalence and incidence [19, 20]). Out of 5392 blood spot samples collected, 2000 were randomly chosen for the immunoassay and after quality control, 1931 samples were used for data analysis. Blood spot filter papers were prepared by punching two discs of 4-mm diameter, and eluted overnight in 160 μL of PBS-TBN (dilution 1:40, PBS-1 % BSA-0.15 % Tween, pH 7.4, Sigma-Aldrich). Just before use in the immunoassay, the eluted samples were further diluted to 1:200 in PBS-CR.

### Antigens

**Fig. 1:**
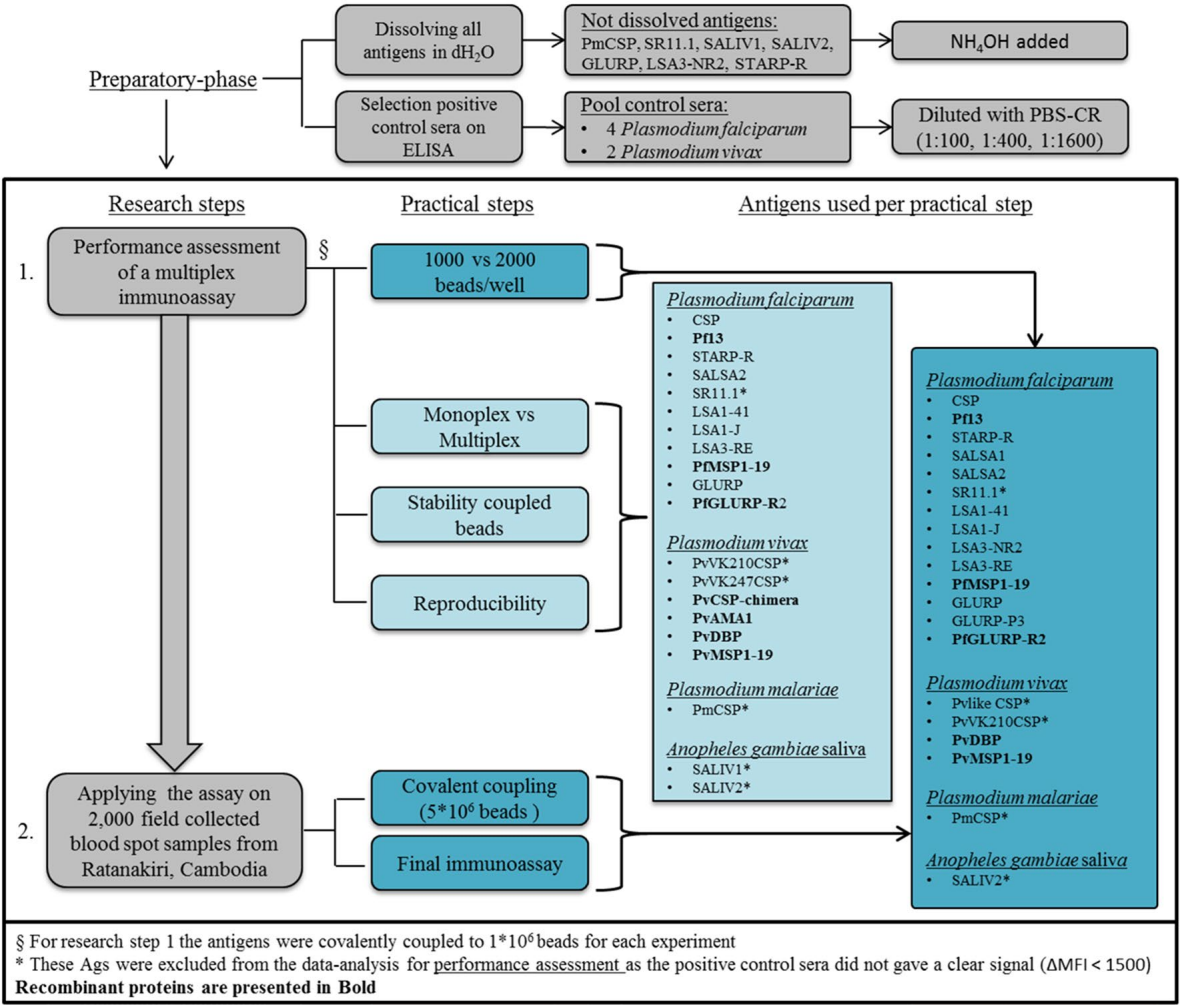
Overview of the study, indicating the antigens used in each step

Selection of peptides specific for *P. falciparum* representing different life stages of the parasite was based on the work of Ambrosino et al. [5]. Additionally, peptides specific for *Anopheles gambiae* saliva protein [5], *P. vivax* and *Plasmodium malariae* were included in the assay, as well as specific recombinant proteins for *P. falciparum* and *P. vivax* (Table 1). All peptides were chemically synthesized with an added N-terminal cysteine residue and bovine serum albumin (BSA) (Table 1) [5] by GeneCust Europe (Dudelange, Luxembourg). The recombinant proteins were synthesized as described in Table 1. This study consisted of two phases (performance assessment of the assay, and application to field samples; Fig. 1). For practical reasons, some steps carried out during the performance assessment used a slightly different Ag set (Fig. 1).

**Table 1:** Overview of the antigens (peptides and recombinant proteins) used in this study

Antigens	Sequence (N-terminal to C-terminal)	g/mol	Life-cycle stages	*Plasmodium* species	Peptide or recombinant protein	References
CSP	NANPNANPNANPNANPNVDPNVDPC	2557.67	Sporozoite	*P. falciparum*	Peptide	[5]
Pfl3	C-terminal His-tag produced in *E. coli*		Sporozoite	*P. falciparum*	Recombinant protein	[45, 46]
STARP-R	STDNNNTKTISTDNNNTKTIC	2299.42	Sporozoite and liver stage	*P. falciparum*	Peptide	[5, 47]
SALSA 1	SAEKKDEKEASEQGEESHKKENSQESAC	3123.24	Sporozoite and liver stage	*P. falciparum*	Peptide	[5, 47]
SALSA 2	NGKDDVKEEKKTNEKKDDGKTDKVQEKVLEKSPKC	4019.52	Sporozoite and liver stage	*P. falciparum*	Peptide	[5, 47
SR11.1	EEVVEELIEEVIPEELVLC	2213.5	Sporozoite and liver stage	*P. falciparum*	Peptide	[5, 47]
LSA1-41	LAKEKLQEQQSDLEQERLAKEKLQEQQSDLEQERLAKEKEKLQC	5297.97	Liver stage	*P. falciparum*	Peptide	[5, 6, 48]
LSA1-J	ERRAKEKLQEQQSDLEQRKADTKKC	3046.43	Liver stage	*P. falciparum*	Peptide	[5, 47, 48]
LSA3-NR2	VLEESQVNDDIFNSLVKSVQQEQQHNVC	3230.53	Liver stage	*P.falciparum*	Peptide	[5, 47]
LSA3-RE	VESVAPSVEESVAPSVEESVAENVEESVC	2991.2	Liver stage	*P. falciparum*	Peptide	[5, 47]
PfMSPl-19	Glutathione S-transferase (GST) fusion protein. C-terminal expressed in *E. coli*		Merozoite	*P. falciparum*	Recombinant protein	[4, 24]
GLURP	EDKNEKGQHEIVEVEEILC	2241.47	Trophozoite	*P. falciparum*	Peptide	[5, 47, 49]
GLURP-P3	EPLEPFPTQIHKDYKC	1945.23	Trophozoite	*P. falciparum*	Peptide	[5, 47, 50]
PfGLURP-R2	C-terminal produced in *E. coli*		Trophozoite	*P. falciparum*	Recombinant protein	[49]
Pvlike CSP	APGANQEGGAAAPGANQEGGAAAPGANQEGGAAC	2892.99	Sporozoite	*P. vivax*	Peptide	[47]
PvVK210 CSP	DGQPAGDRAAGQPAGDRADGQPAGDRADGQPAGC	3206.3	Sporozoite	*P. vivax*	Peptide	[47, 51, 52]
PvVK247 CSP	ANGAGNQPGANGAGNQPGANGAGNQPGANGAGNC	2905.95	Sporozoite	*P. vivax*	Peptide	[47, 51, 52]
PvCSP-chimera	Soluble His-tag protein expressed in wheat-germ cell free expression system		Sporozoite	*P. vivax*	Recombinant protein	[51, 52]
PvAMAl			Merozoite	*P. vivax*	Recombinant protein	[53, 54]
PvDBP			Merozoite	*P. vivax*	Recombinant protein	[55]
PvMSPl-19	C-terminal produced in the baculovirus expression system		Merozoite	*P. vivax*	Recombinant protein	[4, 16, 24]
PmCSP	GNAAGNAAGNDAGNAAGNAAGNAAGNAAGNAAC	2358.37	Sporozoite	*P. malariae*	Peptide	[47]
SALIV 1	EKVWVDRDNVYCGHLDCTRVATFC	2830.22	Salivary gland proteins	*An. gambiae*	Peptide	[5, 56]
SALIV 2	ATFKGERFCTLCDTRHFCECKETREPLC	3324.84	Salivary gland proteins	*An. gambiae*	Peptide	[5, 56]

Ags are organized according to the Plasmodium species and the life-cycle stages in the human host

### Covalent coupling of antigens to the beads/microspheres

Covalent coupling of paramagnetic beads (MagPlex microspheres, Luminex Corp, Austin, TX, USA) was carried out as described by Ambrosino et al. [5] and the Luminex Corp [21, 22]. Each Ag was coupled at a concentration of 4 μg Ag/10^6^ beads to 1 × 10^6^ beads/beadset for performance assessment (Fig. 1). When applying the assay on blood samples from Ratanakiri, all Ags were coupled twice to 5 × 10^6^ beads/beadset, and mixed for homogenous coupling. BSA (Sigma-Aldrich, St Louis, USA) was coupled to an additional set of beads to serve as a background control [5, 23].

### Bead-based immunoassay

The immunoassay was carried out as described previously [5, 17, 22, 24, 25], with minor adjustments. A microsphere working mixture was prepared in PBS-CR, with a concentration of 1000 beads/Ag/well (except in the experiment to assess the difference between a concentration of 2000 and 1000 beads/Ag/well). For the monoplex assay the microsphere working mixture consisted of only one beadset with a coupled Ag, whereas it consisted of a pool of all coupled beadsets for the multiplex assay. In a 96-well plate 25 μl of the microsphere working mixture (at 40 beads/μl) was added per well and 50 μl of serum sample (1:200 dilution) [24]. Plates were incubated at room temperature in the dark for 1 h on a plate shaker (600 rpm). Plates were washed three times, and 100 μl/well of secondary antibody (R-phycoerythrin^+^-conjugated AffiniPure F(ab′)_2_ fragment of goat anti-human IgG, Jackson Immuno Research Laboratories) at a dilution of 1:500 was added [24]. Plates were incubated for 30 min in the dark at room temperature, and washed three times. Beads were resuspended in 100 μl of 5 % PBS-BSA, pH 7.4, and read by the MAGPIX^®^ system. A minimum of 400 beads per spectral address were analysed and results were expressed as the median fluorescence intensity (MFI) [5, 25, 26].

### Experiments to assess the performance of the multiplex immunoassay

For performance assessment, two bead concentrations of 1000 and 2000 beads/Ag/well were compared. The assays performed in monoplex (each Ag separately) and multiplex (all Ags pooled) were compared. Both assays were carried out in triplicate on the positive control pool dilutions (1:100, 1:400, 1:1600) as previously described [5, 25].

Stability of the Ag coupled beads was assessed by performing a multiplex assay directly after coupling the beads and after storage at 4 °C during two, three, four, eight, and 16 weeks.

Reproducibility of the beads coupling was tested by coupling all Ags at three different time points, followed by an immunoassay on positive control pool dilutions (inter-coupling variability) [17]. Inter- and intraplate reproducibility of the immunoassay was assessed by performing the assay on the positive control pool dilutions on three different plates at different time points in 1 day. This was carried out on three different days (interplate variability) and in each plate the positive control pool dilutions were analysed in six-fold (intraplate variability) [27]. In addition, the inter- and intraplate variability was assessed by looking at the positive control pool samples added to each plate with the field blood samples.

### Analysis of field samples

The immunoassay was applied on 2000 field blood samples. Therefore, the microsphere working mixture was prepared at a final concentration of 1000 beads/Ag/well. All field blood spots were analysed in duplicate on separate plates. In each plate, the positive control pool dilutions (1:100, 1:400, 1:1600), negative control serum (1:200) and blanco (PBS-CR) were analysed in duplicate. In total, 50 plates were analysed and read by the MAGPIX^®^ system.

### Data analysis

All data were incorporated and analysed with R software package version 3.1.0. [28]. Results were corrected for background signal by subtracting the signal obtained with BSA-coupled beads (MFI_BSA_) to the median value of the Ag-coupled beads (MFI_Ag_), defined by: ΔMFI = MFI_Ag_ − MFI_BSA_ [25].

### Data analysis for performance assessment

Ags of which the positive control sera did not give a clear signal (ΔMFI < 1500) were excluded from the data-analysis for performance assessment. Non-parametric Spearman’s rank correlation tests were used to analyse the relation between the ΔMFIs for comparing 2000 vs 1000 beads/Ag/well, as well as the monoplex versus the multiplex assay. Correlations were considered significant at p <0.05. Segmented regression models were used for assessing the stability of the beads over time (R package ‘segmented’ [28]). First the breakpoint per Ag and per dilution was estimated, followed by choosing the best linear model through ANOVA tests. Ags showing similar breakpoints in time were grouped and the segmented regression model was rerun on the grouped Ags, taking into account the Ags and dilutions as factor. Mean, standard deviation (SD) and relative standard deviation [RSD $\left( {\frac{SD}{Mean} \times 100\,\% } \right)$] expressed as a percentage of the mean were calculated from the ΔMFI for assessing the reproducibility of the assay.

### Quality control of the multiplex assay when applied on field blood samples

To assure the validity of the plates for screening the field blood samples, a quality control was performed on the ΔMFI values of the high positive control pool samples and on the percentage positivity (PP) calculated from the low positive control pool samples. Results were plotted in Levey Jenning Charts (see Additional file 1) and plates with samples that fell out of −2SD and +2SD were rejected and repeated. After quality control, ΔMFI values of each sample and its duplicate were normalized for each Ag using the value of the high positive control (which was in the linear range of the assay). For this purpose, ΔMFI was converted to PP ($\frac{{\varDelta {\text{MFI}} {\text{samples}} (Ag1)}}{{\varDelta {\text{MFI High positive control}} ({\text{Ag}}1)}} \times 100\, \%$) and then the mean PP of the two duplicate samples was calculated. For each sample, the result was rejected when the RSD (relative standard deviation) of two duplicate results exceeded 30 %. The results of a total of 1931 samples were accepted for further analysis.

### Estimation of the force of infection per antigen

To estimate the seroprevalence per Ag, a cut-off value per Ag was generated by fitting a normal mixture model on the transformed data using the natural logarithm of (PP + 1) [4]. The mean of the negative distribution +3SD was defined as threshold for seropositivity [4]. These dichotomized serological results were used to fit a simple reversible catalytic conversion model based on maximum likelihood. This model estimates one seroconversion rate (SCR, λ) that represents the force of infection and one seroreversion rate (SRR, ρ) per Ag for all individuals [4, 7, 9], expressed per person per year. Next, a model estimating two SCRs and one fixed SRR varying across different age groups was fitted. The breakpoint in age was selected using V-fold cross-validation (VFCV) [10]. VFCV randomly partitioned the data into a validation- and training-set. The cross-validation process was repeated five (V) times on the training set and at least once on the validation set. All V results were averaged to one single value (breakpoint). Partitioning the data avoids overfitting [10, 29]. Both models were compared through the likelihood ratio test (LRT) at p <0.05 [10, 30].

### Ethical clearance

The study protocol was reviewed and approved by the Cambodian National Ethics Committee on Health Research (Approval 265 NECHR), the Institutional Review Board of the Institute of Tropical Medicine Antwerp (Approval IRB/AB/ac/154) and the Ethics Committee of the University of Antwerp (Approval B300201112714). Gatekeepers provided informed written consent for the participation of their village. The survey participant or his/her parents or guardian provided informed written consent for individual participation.

## Results

No clear ΔMFI signal was found for the positive control sera for Ags SR11.1, PvlikeCSP, PvVK210CSP, PvVK247CSP, PmCSP, SALIV1 and SALIV2 (ΔMFI < 1,500). These antigens were therefore excluded from further analysis in the performance assessment (Fig. 1).

### Performance assessment of the multiplex immunoassay using 1000 performs as good as 2000 beads/Ag/well

**Fig. 2:**
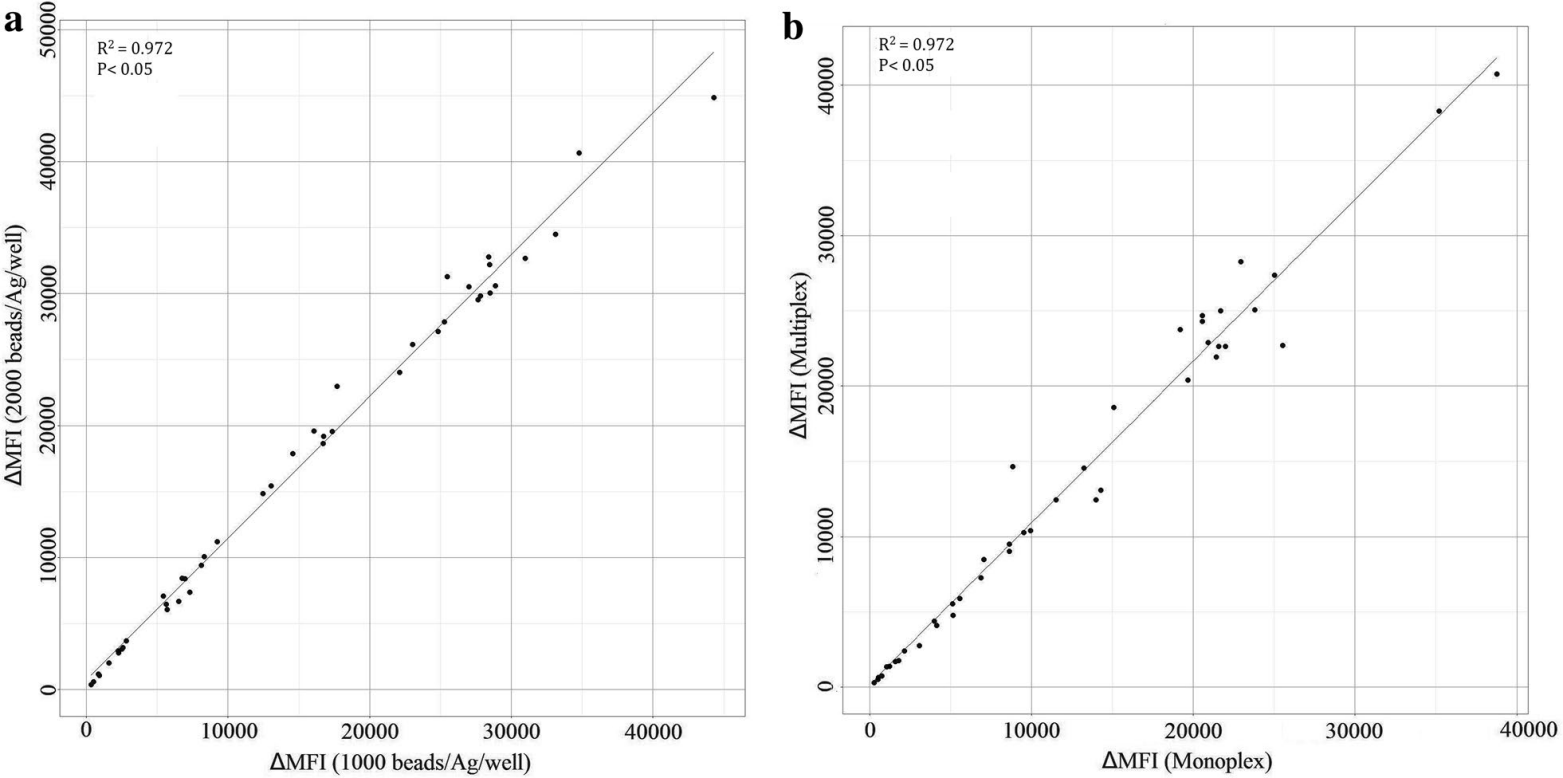
Correlation between the ΔMFI values obtained from analysing the positive control pool with the bead-based assay **a** using 1000 and 2000 beads/Ag/well, and **b** in the monoplex and multiplex format

The ΔMFIs obtained from the assays using 1000 and 2000 beads/Ag/well were significantly correlated (Spearman’s Rank correlation p < 0.05; R^2^ = 0.972; Fig. 2a). For most Ags, a slightly higher ΔMFI was observed when using 1000 beads/Ag/well as compared to 2000 beads/Ag/well (Additional files 2, 3), and this for all dilutions of the positive control pool (1:100, 1:400 and 1:1600).

### Multiplexing beads does not affect the assay results

Similar ΔMFI-signals were obtained in monoplex and multiplex (Additional files 2, 3), with a clear correlation for all Ags between the ΔMFIs obtained by the monoplex and the multiplex assays (R^2^ = 0.972; p < 0.05; Fig. 2b).

### Stability of the coupled beads depends on the antigens used

**Fig. 3:**
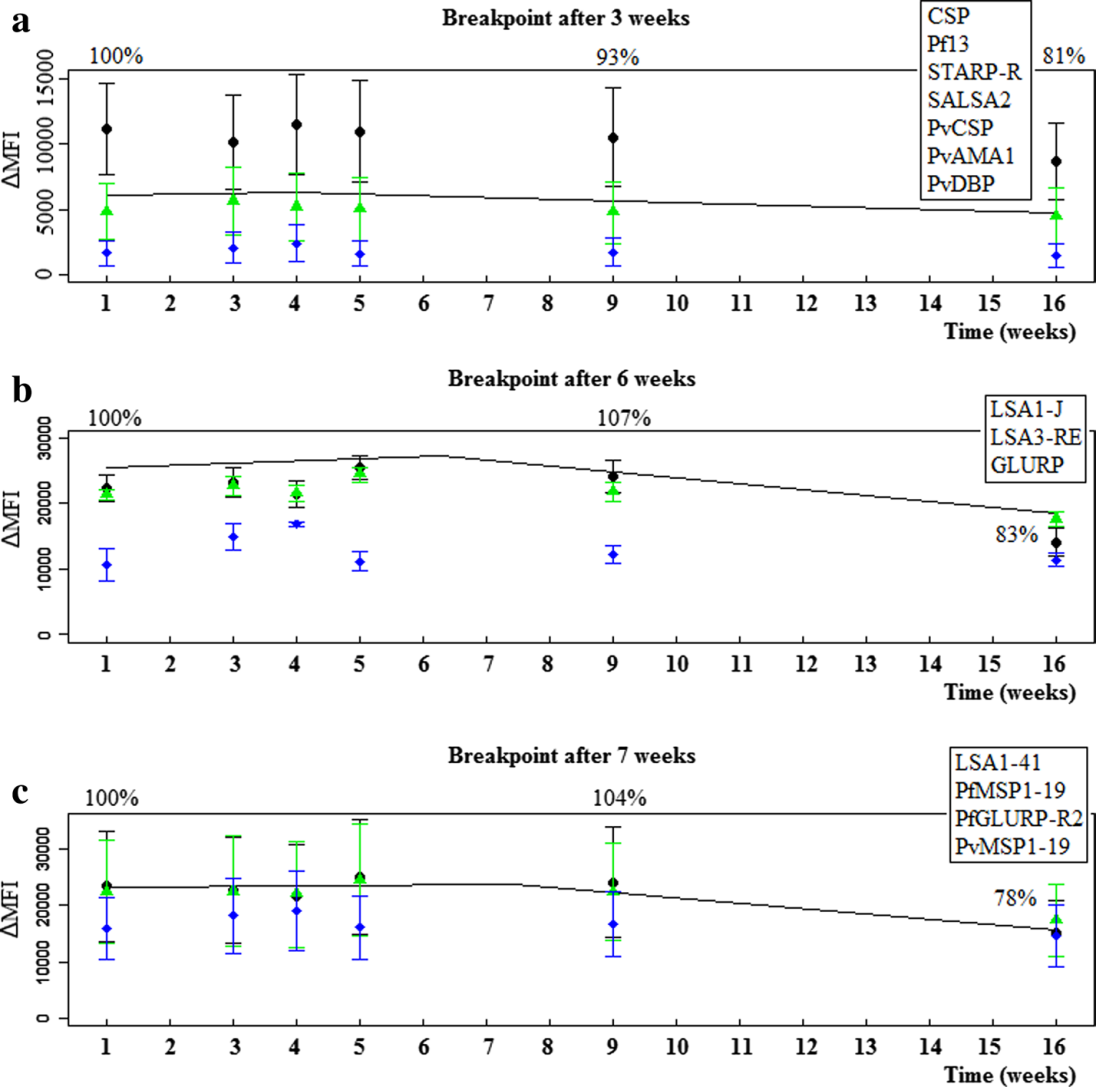
The stability of the coupled beads as determined by segmented regression analysis taking into account antigens and dilutions as factor. Beads were tested at different time points after coupling and the breakpoints in time were estimated by segmented regression analysis. The trend in ΔMFI shows different breakpoints for three groups of antigens, namely **a** 3 weeks after coupling (CSP, Pf13, STARP-R, SALSA2, PvCSP, PvAMA1, and PvDBP), **b** 6 weeks after coupling (LSA1-J, LSA3-RE, GLURP) and **c** 7 weeks after coupling (LSA1-41, PfMSP1-19, PfGLURP-R2, and PvMSP1-19)

Segmented regression models distinguished three groups of Ags, each with a similar ΔMFI trend over time (Fig. 3). For these three groups, different breakpoints (i.e., points in time at which the trend in ΔMFI changes) were observed at 3, 6 and 7 weeks after coupling. For the Ags with lower ΔMFI values (CSP, Pf13, STARP-R, SALSA2, PvCSP, PvAMA1, and PvDBP) an earlier break in trend is observed, but with a low ΔMFI decay over time after the breakpoint (Fig. 3; Additional file 4). In general, for all Ags except for PvCSP, the decay in ΔMFI after 8 weeks was less than 10 % (Additional file 4). As such, the coupled beads can be used for at least 8 weeks after coupling.

### The immunoassay is reproducible

**Fig. 4:**
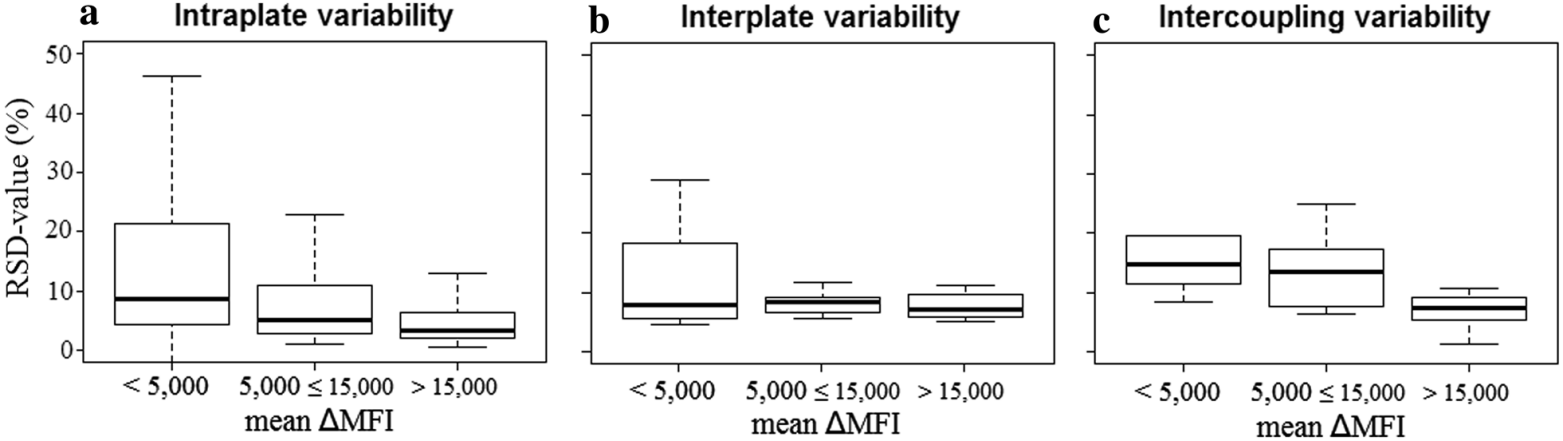
Reproducibility of the bead coupling and immunoassay determined experimentally by analysing the positive control pool. The relative standard deviation (RSD, y-axis) is plotted in relation to the mean ΔMFI values (x-axis) obtained from the assay. The *boxplots* represent the 75th percentile, median and 25th percentile of the RSD values from the **a** intercoupling-, **b** intraplate and **c** interplate variability per dilution (1:100, 1:400 and 1:1600). *Whiskers* represent the maximal and minimal outlier limits

**Fig. 5:**
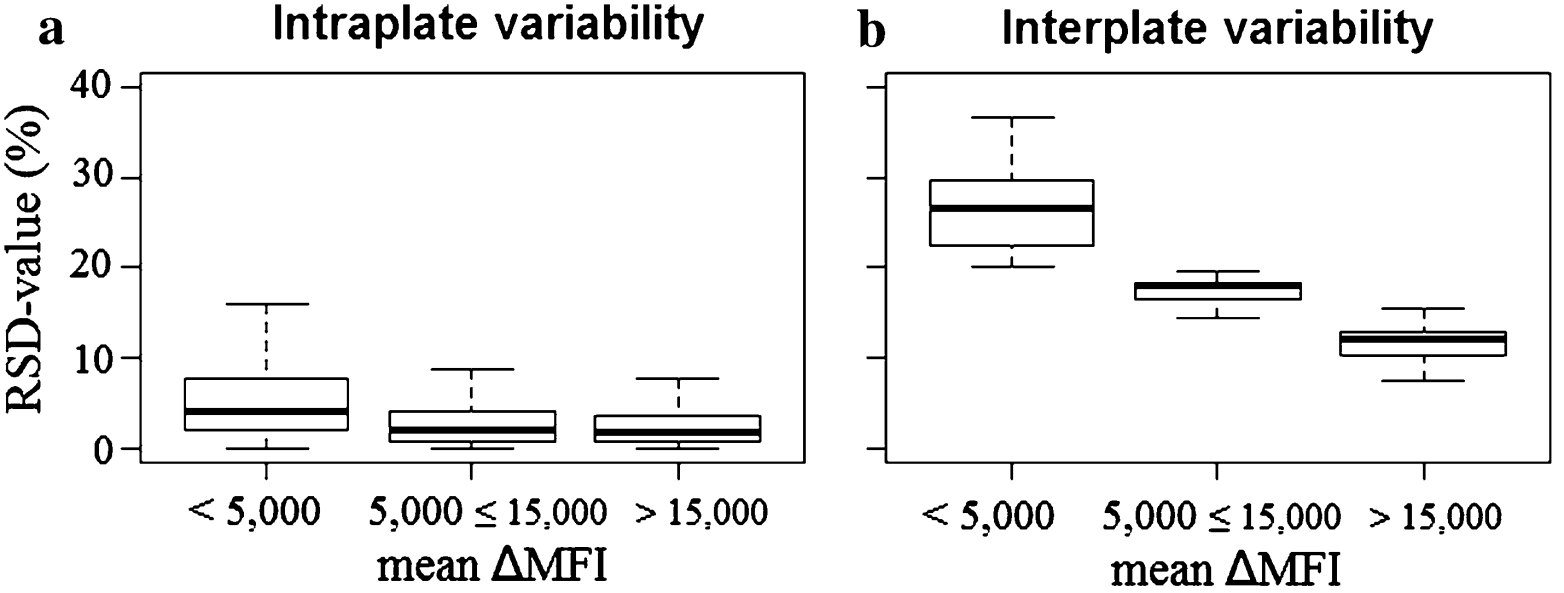
Reproducibility of the immunoassay based on the quality control samples in the immunoassay applied on field collected samples. The relative standard deviation (RSD, y-axis) is plotted in relation to the mean ΔMFI values (x-axis) obtained from the assay. The *boxplots* represent the 75th percentile, median and 25th percentile of the RSD values from the intercoupling, intraplate and interplate variability per dilution (1:100, 1:400 and 1:1600). *Whiskers* represent the maximal and minimal outlier limits

Intercoupling, intraplate and interplate reproducibility was assessed per Ag by evaluating the RSD values (Fig. 4). The ΔMFI results were divided into three different groups (<5000; 5000; ≤15,000; >15,000). Highest RSD values were observed for the lowest ΔMFI values (<5000). In general, the upper limits of the interquartile ranges were lower than 15 % [inter-coupling 11.9 % (8.5–15.2 %), interplate 7.9 % (6.1–12.4 %), intraplate 5.7 % (3.0–12.7 %)] which indicates an acceptable reproducibility [27]. The intraplate and interplate variability was also assessed by analysing the RSD on the ΔMFI of the high, medium and low positive control pool dilutions used as a quality control on the 50 plates with field blood samples (Fig. 5). A low variability was observed for the intraplate reproducibility [RSD 2.7 % (IQR: 1.2–5.1 %)]. The interplate variability was higher than experimentally determined during the performance assessment [RSD 18.6 % (IQR: 16.5–20.1 %)], but still acceptable.

### Application of the assay on blood samples collected in Ratanakiri

#### Quality control of the high positive control serum

A total of 2000 field blood samples were analysed in duplicate in a total of 50 96-well plates. These plates were validated based on the Levey Jenning Charts of the ΔMFI of the high positive controls and the PP of the low positive controls (Additional file 1). The stability of the ΔMFI signal of the positive control pool over the analysis of the plates was confirmed by segmented regression, as no breakpoint could be found for the Ags. Based on the duplicate results of the field blood samples, the results of 1931 blood samples were validated for further analysis.

#### Estimating age-group specific seroconversion rates per antigen

**Fig. 6:**
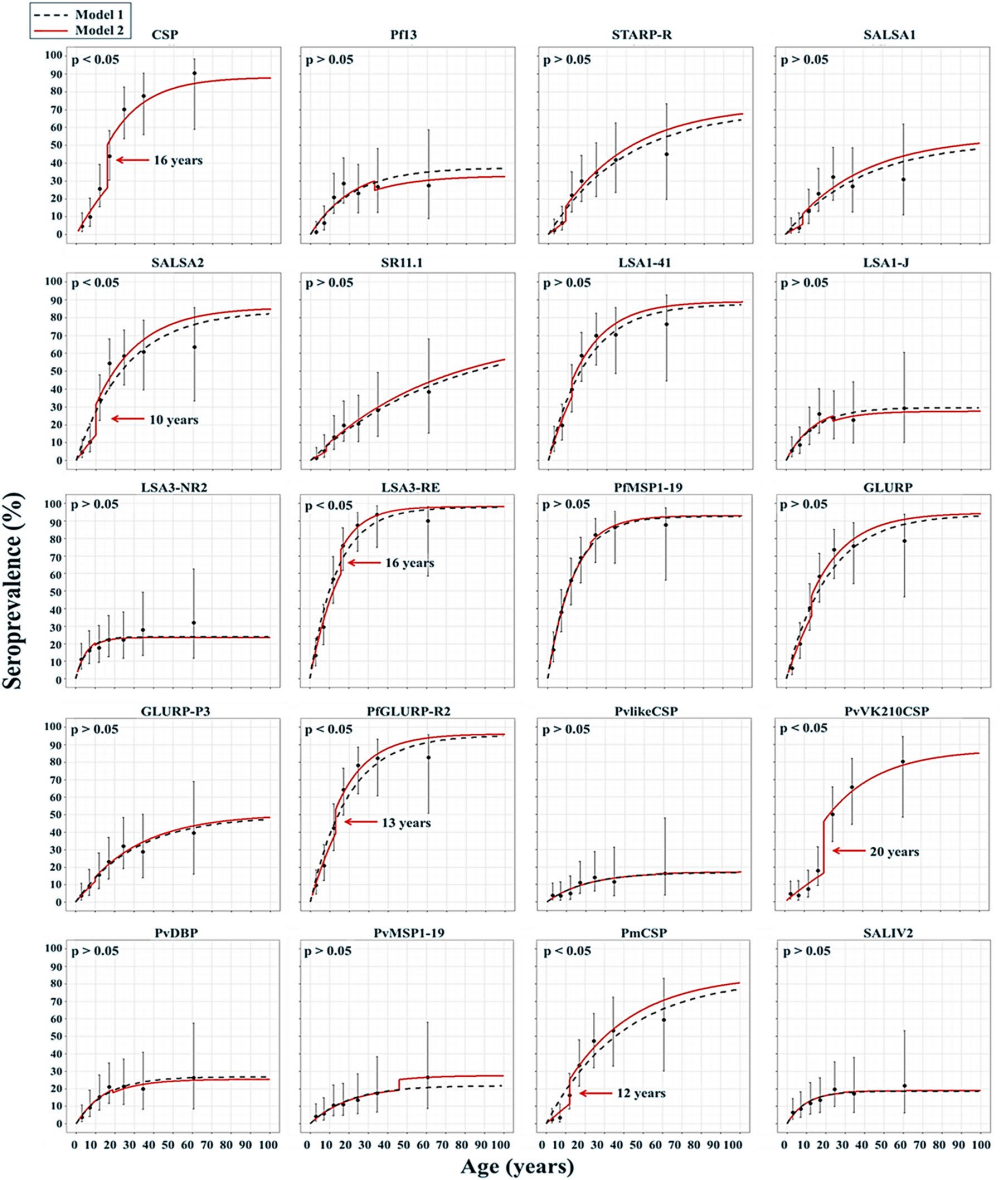
Age-seroprevalence curves for all antigens analysed by the multiplex assay. Reversible catalytic conversion models allowing one (*dashed black line*, Model 1) and two (*red solid line*, Model 2) seroconversion rates were fitted to the data. For six Ags the model allowing two SCRs fitted better as compared to one SCR

**Fig. 7:**
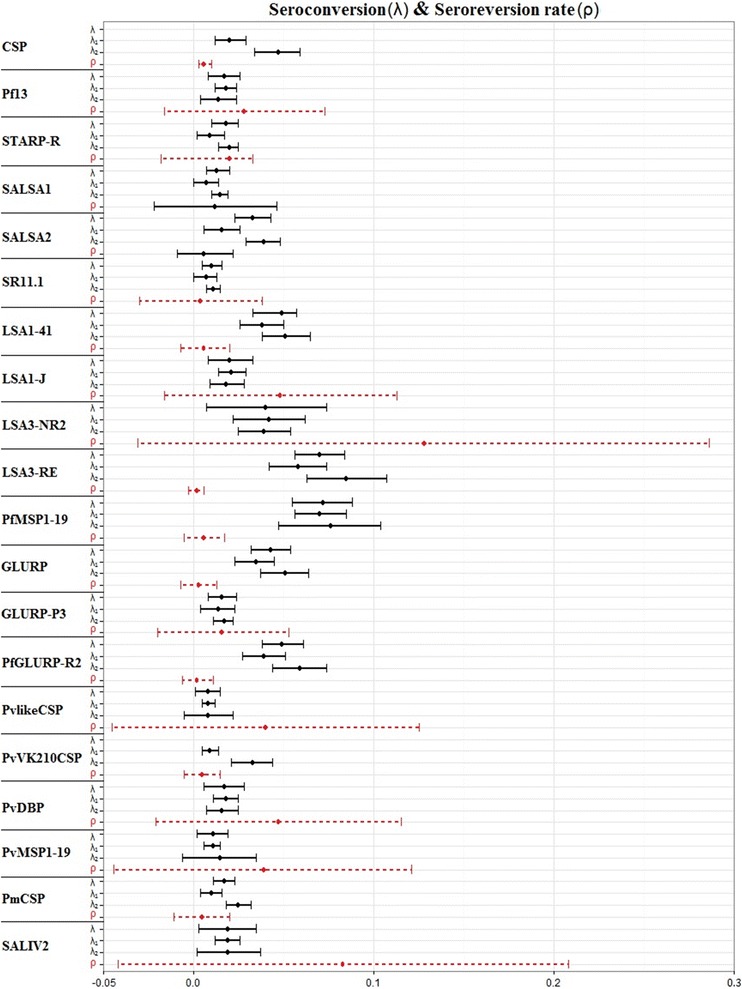
Comparison of seroconversion and seroreversion rates between serological markers. The estimated SCRs of reversible catalytic conversion models allowing one (λ) and two (λ1 and λ2) SCRs are plotted with their 95 % confidence intervals. The SRRs are obtained from the model allowing one SCR. Antigens are sorted per parasite and life stage

After dichotomizing the serological results for all blood samples based on a cut-off value, age-seroprevalence curves were constructed and reversible catalytic conversion models allowing one and two SCRs were fitted to the data (Fig. 6; Additional file 5). The force of malaria infection between age groups was shown for 6 out of 20 Ags (CSP, SALSA2, LSA3-RE, PfGLURP-R2, PvVK210CSP, and PmCSP), with the time of change obtained through V-fold cross-validated plots (Additional file 6) ranging between 10 and 20 years. Higher SCRs were observed for the higher age groups for these Ags. When comparing the SCRs and SRRs between serological markers used (Fig. 7), large differences were observed among serological markers used for different life stages of the same parasite, as well as for different parasites. Within the population, highest annual SCRs were modelled for Ags LSA3-RE (0.085 per person per year) and PfMSP1-19 (0.076 per person per year) and lowest annual SCRs for Ags SALSA1, SR11.1, PvlikeCSP, STARP-R, and PvVK210CSP (ranging from 0.007 to 0.009 per person per year). Highest annual SRRs were modelled for Ags LSA3-NR2 (0.128 per person per year) and SALIV 2 (0.083 per person per year), and lowest for Ags LSA3-RE, PfGLURP-R2, GLURP, SR11.1, PvVK210CSP, and PmCSP (ranging from 0.002 to 0.005 per person per year).

## Discussion

This is the first implemented multiplex assay for malaria serology in Southeast Asia. Moreover, with the use of 20 specific Ags against different *Plasmodium* parasites (Table 1), this assay is the most extensive multiplex assay published on malaria serology, besides Helb et al. [32]. In contrast to most studies, the Abs that can be detected are not only directed against one or two different *Plasmodium* parasites [5, 10, 17, 24, 25, 33], but responses against *P. falciparum*, *P. vivax*, *P. malariae*, and *An. gambiae* are simultaneously detected. This is important in malaria elimination programmes, and especially in Southeast Asia where all four human malaria parasites as well as *Plasmodium knowlesi* are co-occurring [34]. Unfortunately at this stage, Ags specific for *Plasmodium ovale* and *P. knowlesi* were not available and thus not included in the assay. As the final aim of the study was to analyse 2000 field blood samples, the stability of the coated beads over time and the reproducibility of the assay were first thoroughly assessed and proved to be satisfactory.

Previously it has been shown that multiplex-based serological studies are useful for detecting anti-*Plasmodium* Abs [5, 10, 15, 17, 18, 24, 25] as well as Abs against other pathogens [31, 35–38]. Also in the present study, the technique was shown to be reliable and fast in detecting several serological markers at once. All samples were analysed in less than 8 weeks of time, and more recently, over 8000 samples were analysed in duplicate in the same timeframe, showing the possibility to upscale the sample throughput. However, complexity, high investment costs in purchasing a Luminex Analyzer and need for special training, prevent widespread use as compared to ELISA [5, 6]. A major restriction of this study is the use of the pool of control serum, as this has to be the same during the entire study, adequate quantities should therefore be prepared and stored in advance. In previous studies, satisfactory evidence has been presented on the validation of bead-based in comparison to ELISA [5, 39], and this was, therefore, not further explored in the present study.

Several features were considered to assess the performance of the assay. In accordance to previous observations [17], this study confirmed that a reduction of the beads to 1000 beads/Ag/well leads to similar results as compared to the amount of beads recommended by the standard protocols for Luminex-based multiplex immunoassays (2000 or 5000 beads/Ag/well). A rough cost estimation indicates that the use of 1000 instead of 2000 beads/Ag/well reduces the assay costs by one-third. Moreover, the results of previous studies [5, 15, 17] in which monoplex assays resulted in highly comparable ΔMFI values as multiplex assays were confirmed. This refutes the concern on cross-reactivity between the different Ag-coupled beads or blocking of Ab responses [15].

The obtained results of the performance assessment revealed that most of the coated beads were stable for up to 2 months, except for Ag PvCSP. This is important as screening approximately 2000 blood spot samples in duplicate took about 7 weeks from the first coupling until the last immunoassay (based on the results 1143 samples can be analysed per week). This stability is lower compared to previous studies, stating that beads are stable for up to 3 or 7 months [5, 33], except for Ag STARP-R which was previously shown to be stable for only 1 month [5].

Good reproducibility is an important aspect of an assay as it guarantees that results obtained with the assay performed on different plates and at different time points are comparable to each other. In the experimental set-up used in this study, there is no big difference in reproducibility between and within different plates as shown by similar RSDs. This is in line with other studies that showed accurate and repeatable results according to the interplate, intraplate [5, 15, 27, 31], and intercoupling [40] reproducibility of multiplex assays. In this study, the reproducibility was also assessed by looking at the results obtained from the positive control pool dilutions analysed in duplicate on each of the plates used for screening 2000 blood samples from Ratanakiri Province (total of 50 plates). A much smaller intraplate variability (RSD <3 %) was observed compared to the experimental set-up, which was probably due to the fact that samples were only tested in duplicate instead of six times in the experimental set-up. A higher interplate variability was observed, which was still acceptable (RSD <25 %) [27], and most probably due to the use of only two positive controls per plate.

In the present assay the use of peptides and recombinant proteins was combined. Fourteen *Plasmodium*-specific peptides (CSP, STARP-R, SALSA1, SALSA2, SR11.1, LSA1-41, LSA1-J, LSA3-NR2, LSA3-RE, GLURP, GLURP-P3, PvlikeCSP, PvVK210CSP, and PmCSP), one peptide specific for the *An. gambiae* saliva protein (SALIV2), and five *Plasmodium*-specific recombinant proteins (Pf13, PfMSP1-19, PfGLURP-R2, PvDBP, and PvMSP1-19) were included in the immunoassay.

After assessment of its performance, the multiplex assay was applied to blood spot samples collected in Ratanakiri Province in Cambodia to detect Ab responses against 20 different Ags. The seroconversion rate (SCR) was then estimated per Ag by constructing age-seroprevalence curves based on a threshold approach. This is currently the most frequently used way of analysing serological data in malaria epidemiology [4, 7], but which until the present was not applied to data from multiplex assays. By fitting models allowing two SCRs [10], age groups exhibiting similar SCRs can be defined. For six out of 20 Ags (CSP, SALSA2, LSA3-RE, PfGLURP-R2, PvVK210CSP, and PmCSP), the models allowing two SCRs fitted better, and a higher SCR was observed in the older age group. This observation corroborates results obtained in previous studies in Cambodia using GLURP and MSP1-19 as serological markers [9]. A lower SCR in the younger age groups may indicate a lower malaria exposure in the age group. This can be attributed to reductions in malaria transmission over time because of intensified malaria control interventions in the last years, or to different risk behaviour linked to the age groups [9]. Moreover, another explanation would be that children might lose their malaria-specific Abs faster [9]. Indeed, previous studies on malaria serology have shown that Ab responses in adults fluctuate to a lesser extent than in children [41, 42]. The statistical model fit used to obtain the estimates for the serological parameters was constrained to have two constant (i.e. age-independent) SCRs and one constant sero-reversion rate (SRR). More elaborate model fitting incorporating age-dependent SCRs and SRRs fell outside the scope of this study.

Until the present very limited information was available on the half-life of the Ab responses to the Ags used and on the individual variation in those responses. This is crucial for interpreting the results obtained with this assay. Serological markers with a long half-life can be used for assessing past exposure, while a short half-life will be useful for assessing recent or even present exposure. The present study has obtained information on the Ab half-life estimated from the SRR as modelled in the reverse catalytic model [43]. Based on this information, fluctuations in Ab half-life were observed per Ag, but all of them seem to have a relatively long half-life (ranging from 5 to >100 years). Most studies assume a fixed SRR independent of age and transmission rate [30]. A study based on one Ag (PfMSP1-19) [10] compared longitudinal and cross-sectional data (children ages ≤11 years), showing a high concordance of SCRs between both study designs, while large discrepancies were seen between the SRRs. A follow-up study based on a selected combination of Ags (10 out of 655) that compared Ab-responses at individual and population level also shows reliable rates of exposure [32]. The present study is based on cross-sectional sampling and the obtained SRRs are probably not reliable. More extensive analysis on the half-life of these Ab responses is planned by using sequentially collected blood samples from the same individuals, as well as by exploring which of the Ab responses can best capture the fluctuations in malaria prevalence and incidence observed over time. SCR estimates based on a single serological marker can give a good indication of the force of infection [4, 7]. However, combining the signal from multiple markers representing different life-stages of the parasite in an individual could help reducing individually heterogeneous responses to one particular marker, thereby providing more accurate estimation of the force of infection. A recent follow-up study conducted on a cohort of children demonstrated that measuring Ab-responses to a selection of few Ags provide an accurate estimate of exposure for individuals and communities [32]. Therefore, further data analysis performed with multivariate analysis should provide added value. Moreover, presently it is not possible to exploit the full potential of the assay to take into account individual variation in Ab responses, as analysis of the SCR is still carried out Ag by Ag. Data analysis methods to combine Ab responses for estimation of SCRs exist [44] and are currently being extended in the context of this multiplex assay for detection of anti-*Plasmodium* Abs.

## Conclusion

As many countries engage in malaria elimination it is crucial to improve the understanding of the effectiveness of additional malaria control tools and to be able to identify malaria hotspots for targeted and effective interventions in countries in an advanced elimination phase. With the current malariometric and entomological tools it is challenging to determine the malaria transmission rate in areas where prevalence of infection and incidence is decreasing. The multiplex assay measuring serological markers for malaria transmission was successfully implemented as the first in the Southeast Asian context. Moreover, it proved to be a reliable, fast and useful method for simultaneous detection of Abs against *Plasmodium* Ags of three different *Plasmodium* parasites, which has never been done before. The assay was used to screen almost 2000 field blood spot samples in duplicate and proved to be very time efficient and informative. It was also possible to estimate differences in the force of malaria infection, by looking at the differences in SCR over time between the different Ags. For a better estimation of changes in the force of infection on long-term as well as short-term in areas with low malaria endemicity, more information on the longevity of the Ab responses is needed. The simultaneous detection of several Ags of different parasites offers an opportunity to look for a potential serological marker of very recent malaria transmission, which is the next step of this research. This will prove to be key knowledge for malaria elimination.

## Additional files

Additional file 1:
**Example of Levey Jenning Charts plotted for the quality control of the immunoassay used for screening the field bloodspot samples.** Data analysis started with a quality control on the ΔMFI-values of the 100 % positive control pool samples (A). The dots represent each positive control sera sample in duplicate per plate. If these dots fell out of the -2SD and +2SD (red area), these plates were rejected and re-analysed. The same quality control was also performed on the PP calculated from the 50 % positive control pool samples per Ag (B). Based on the outcome of both graphs, plates were accepted or rejected and reanalysed. Additional file 2:
**Comparison of the ΔMFI signals obtained from the immunoassay using 1000 and 2000 beads/Ag/well.** The positive control pool was tested in the multiplex assay (dilutions 1:100, 1:400 and 1:1600) for 1000 beads/Ag/well (dark blue bars) and 2000 beads/Ag/well (light blue bars). The error bars represent upper and lower limits of the 95 % confidence intervals. Additional file 3:
**Comparison of the ΔMFI signals obtained from the monoplex and multiplex immunoassay.** Each Ag was tested in monoplex (each Ag separately) and in multiplex (all Ags pooled together) assay on the positive control pool (dilutions 1:100, 1:400 and 1:1600). The error bars represent upper and lower limits of the 95 % confidence intervals. Additional file 4:
**Results of the segmented regression analysis for assessing the stability of the antigen coupled beads per antigen.** The breakpoints were estimated with the R-package ‘segmented’ and the ΔMFI-values obtained at Week (Wk) 4, Wk 8 and Wk16 were converted into percentages compared to the ΔMFI value obtained directly after coupling (Wk0). Additional file 5: Seroconversion (λ) and seroreversion (ρ) rates estimated for each antigen by catalytic regression models allowing one (Model 1) and two (Model 2) seroconversion rates. Additional file 6:
**V-fold cross-validated plots with age-varying seroconversion rates.** These plots were used to estimate the optimal breakpoint in age per antigen. The VFCV randomly partionate the data into a validation set and a training set. Then the cross-validation is repeated V-times on the training set and a least once on the validation set. All V-results are avaraged and gave a single estimation.
