# Supplementary figures and images for: Implementation and application of a multiplex assay to detect malaria-specific antibodies: a promising tool for assessing malaria transmission in Southeast Asian pre-elimination areas
Source: Malar J. 2015 Sep 4;14:338. doi: 10.1186/s12936-015-0868-z (PMC4558921; doi:10.1186/s12936-015-0868-z)

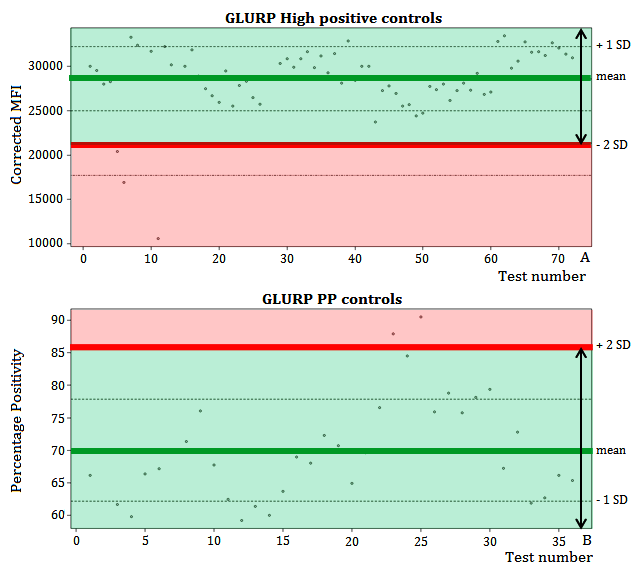

Supplement: Additional file 1: — Example of Levey Jenning Charts plotted for the quality control of the immunoassay used for screening the field bloodspot samples. Data analysis started with a quality control on the ΔMFI-values of the 100 % positive control pool samples (A). The dots represent each positive control sera sample in duplicate per plate. If these dots fell out of the -2SD and +2SD (red area), these plates were rejected and re-analysed. The same quality control was also performed on the PP calculated from the 50 % positive control pool samples per Ag (B). Based on the outcome of both graphs, plates were accepted or rejected and reanalysed. [file 12936_2015_868_MOESM1_ESM.png]

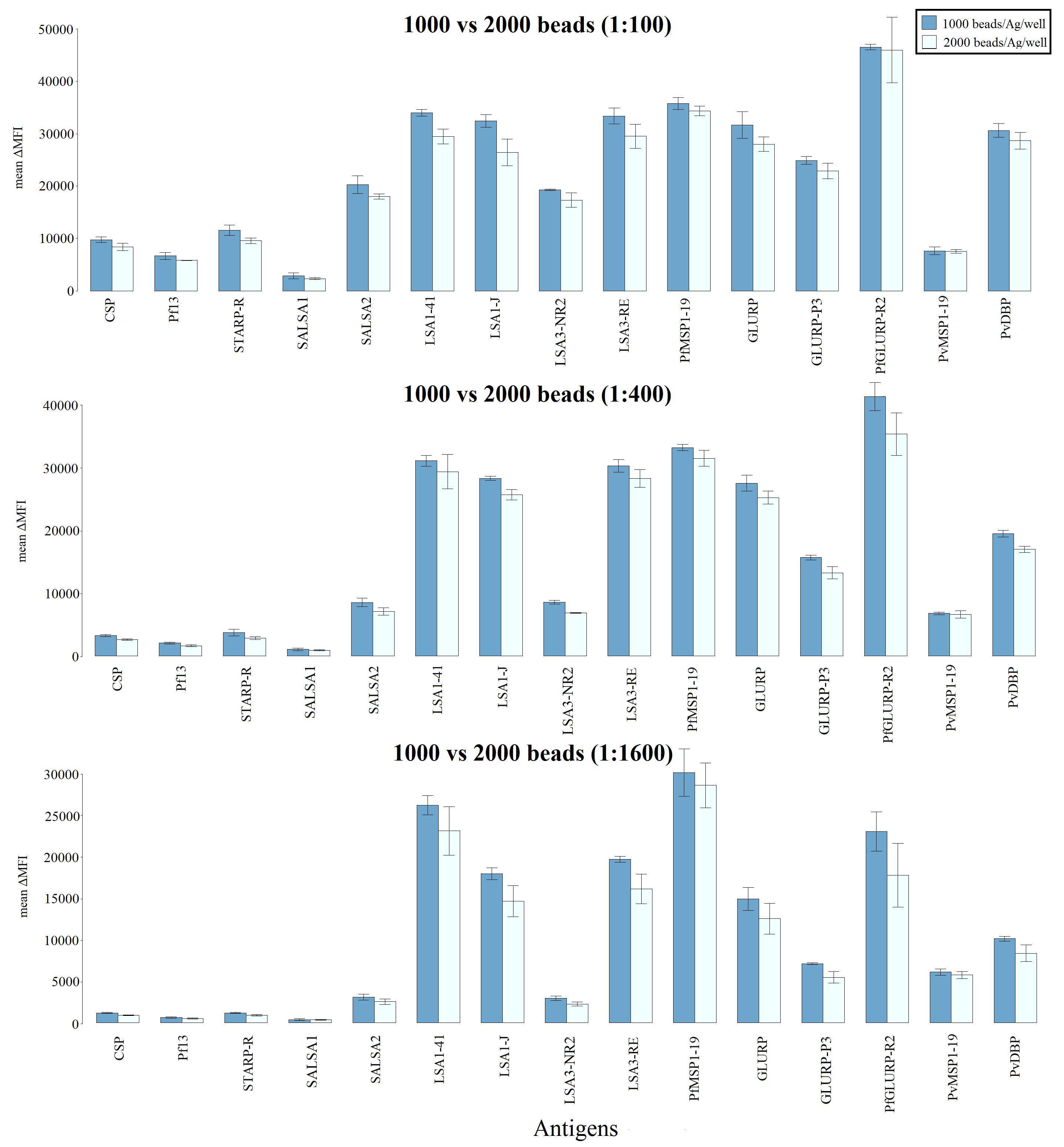

Supplement: Additional file 2: — Comparison of the ΔMFI signals obtained from the immunoassay using 1000 and 2000 beads/Ag/well. The positive control pool was tested in the multiplex assay (dilutions 1:100, 1:400 and 1:1600) for 1000 beads/Ag/well (dark blue bars) and 2000 beads/Ag/well (light blue bars). The error bars represent upper and lower limits of the 95 % confidence intervals. [file 12936_2015_868_MOESM2_ESM.png]

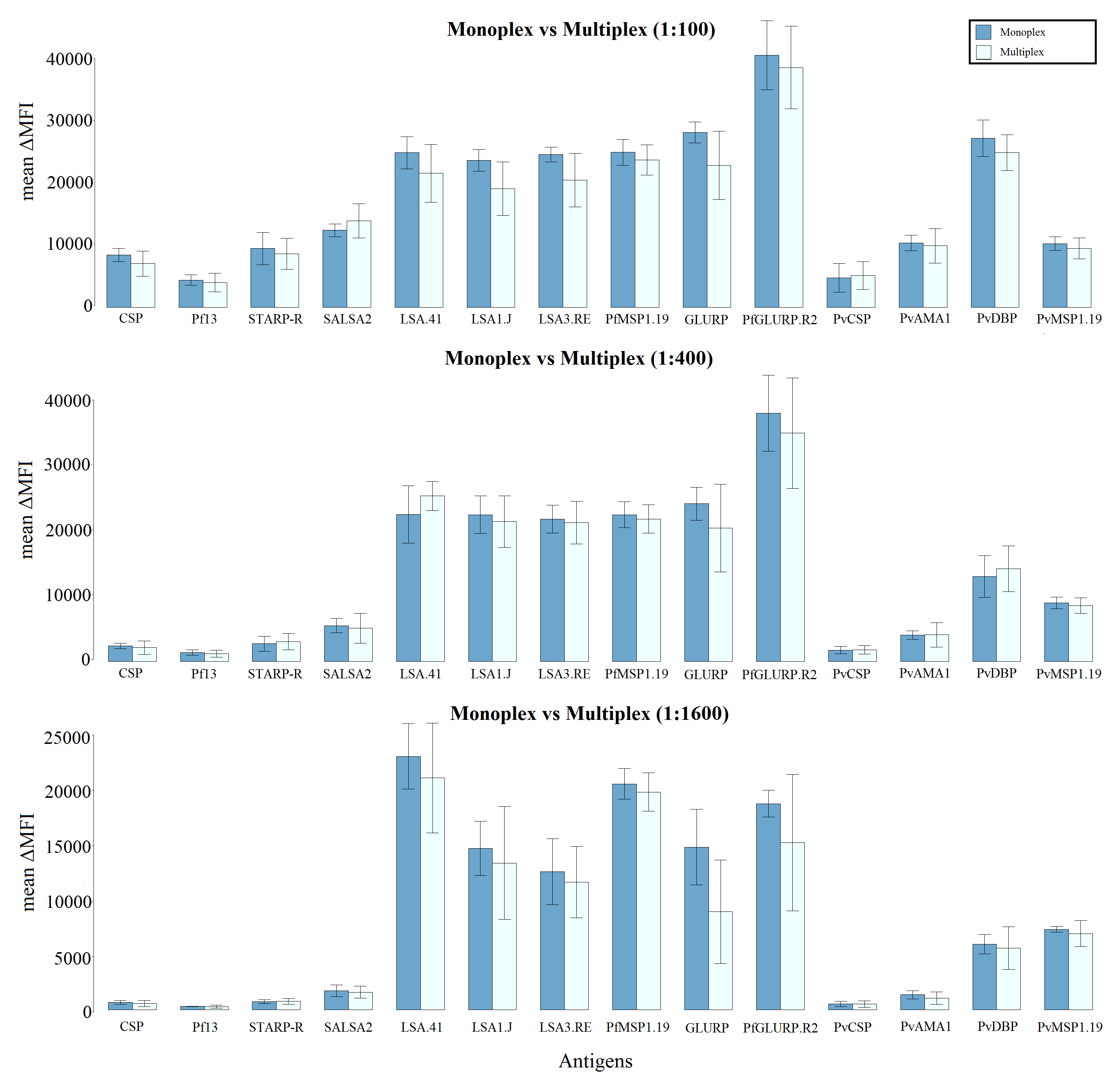

Supplement: Additional file 3: — Comparison of the ΔMFI signals obtained from the monoplex and multiplex immunoassay. Each Ag was tested in monoplex (each Ag separately) and in multiplex (all Ags pooled together) assay on the positive control pool (dilutions 1:100, 1:400 and 1:1600). The error bars represent upper and lower limits of the 95 % confidence intervals. [file 12936_2015_868_MOESM3_ESM.png]

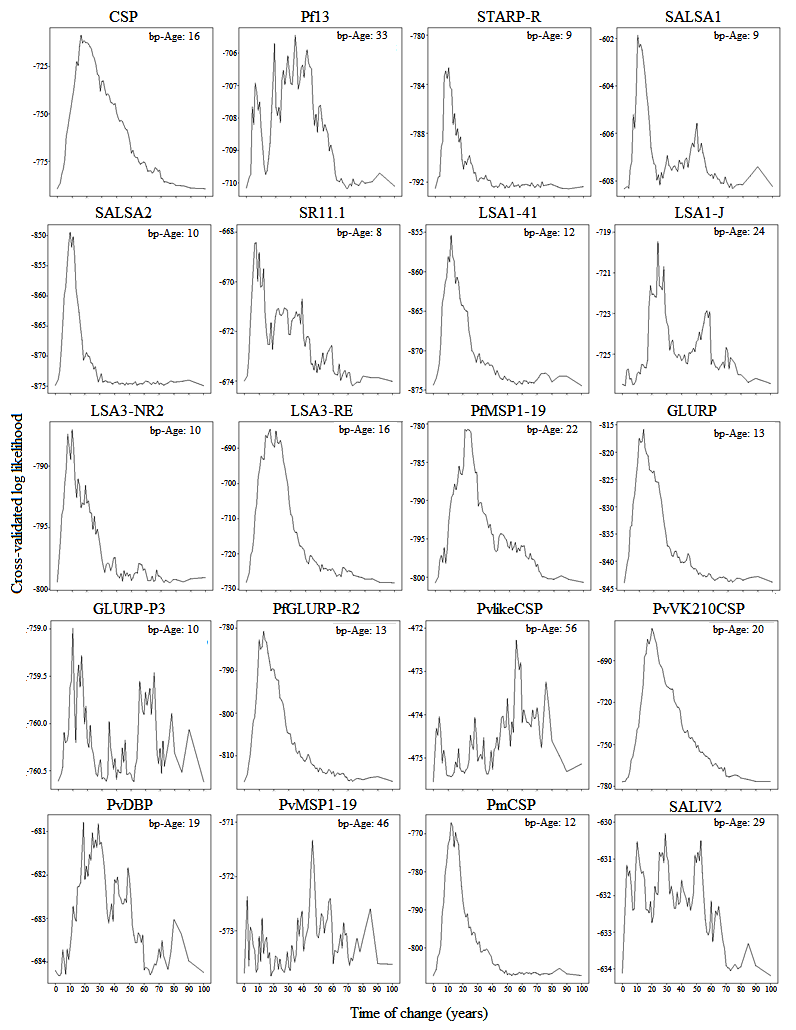

Supplement: Additional file 6: — V-fold cross-validated plots with age-varying seroconversion rates. These plots were used to estimate the optimal breakpoint in age per antigen. The VFCV randomly partionate the data into a validation set and a training set. Then the cross-validation is repeated V-times on the training set and a least once on the validation set. All V-results are avaraged and gave a single estimation. [file 12936_2015_868_MOESM6_ESM.png]
